# Tackling Antimicrobial Resistance: A Sustainable Method for the Removal of Antibiotics from Water

**DOI:** 10.3390/antibiotics14030324

**Published:** 2025-03-19

**Authors:** Lekan Abudu, Rutuja C. Bhosale, Joerg Arnscheidt, Svetlana Tretsiakova-McNally, Barry O’Hagan, David K. Adeyemi, Temilola Oluseyi, Luqman A. Adams, Heather M. Coleman

**Affiliations:** 1 School of Pharmacy and Pharmaceutical Sciences, Ulster University, Coleraine BT52 1SA, UK; lekan.abudu@protonmail.com (L.A.); rutujabhosale997@gmail.com (R.C.B.); 2 Department of Pharmaceutical Chemistry, Faculty of Pharmacy, University of Lagos, Lagos 100272, Nigeria; dadeyemi@unilag.edu.ng; 3 School of Geography and Environmental Sciences, Ulster University, Coleraine BT52 1SA, UK; j.arnscheidt@ulster.ac.uk; 4 Belfast School of Architecture and the Built Environment, Ulster University, Belfast BT15 1ED, UK; s.tretsiakova-mcnally@ulster.ac.uk; 5 School of Biomedical Sciences, Ulster University, Coleraine BT52 1SA, UK; bmg.ohagan@ulster.ac.uk; 6 Department of Chemistry, University of Lagos, Lagos 100272, Nigeria; toluseyi@unilag.edu.ng (T.O.); ladams@unilag.edu.ng (L.A.A.)

**Keywords:** water treatment, antibiotics, sawdust, adsorption, antibiotic resistance, rifampicin

## Abstract

**Introduction**: The presence of antibiotic residues in the aquatic environment is a likely contributor to the current increase in antibiotic resistance, posing a significant threat to global health. This study investigated the use of a low-cost and sustainable material based on sawdust with the purpose of removing rifampicin residues from water. **Methods**: The sawdust was pretreated with 2M sulfuric acid and was characterized using Fourier Transform Infrared spectroscopy (FT-IR), a Mastersizer, scanning electron microscopy (SEM), an elemental analyser, and the pH point of zero charge (pH_pzc_). The batch adsorption process was conducted using both raw and treated sawdust to determine the effect of contact time, temperature, pH, adsorbent dosage, and the initial concentration of antibiotic dissolved in water. **Results and Discussion**: The results revealed that the chemical pretreatment of raw sawdust significantly improved its adsorption capacity. The highest removal efficiency of 65% was achieved using an adsorbent dosage of 31.3 g/L. The thermodynamic studies demonstrated that the process was spontaneous and governed by physisorption within the studied temperature range (293.15 K–318.15 K), being more favourable at higher temperatures. The interactions between the functional groups of sawdust and the rifampicin molecules included electrostatic attraction, hydrogen bonding, and π-π interactions. **Conclusions**: This research highlights the potential of utilizing waste as a valuable and effective adsorbent of residual antibiotics from water, thus contributing to the sustainable practices of solid waste management and water treatment.

## 1. Introduction

**Figure 1:**
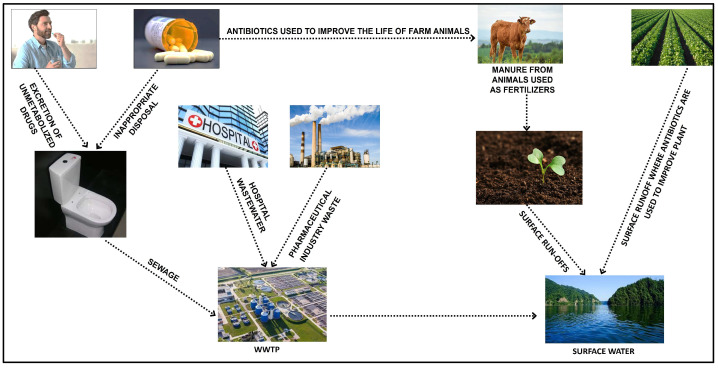
Sources of antibiotic residue in the environment.

For decades, antibiotics—a class of pharmaceuticals used to treat infection—have been detected in our environments, such as in soil and water, due to excessive and improper usage [1]. Their presence, for example in water, poses a threat to human health, even though they are generally designed to eliminate microbes without affecting host organisms. However, the continuous and uncontrolled exposure of microbes to antibiotics, even at low concentrations, has accelerated their acquisition of antimicrobial resistance. This is usually achieved through the transfer of existing genes between strains or even different microorganisms [2]. As shown in Figure 1, one of the major sources of antibiotics in the environment is the effluent of wastewater treatment plants (WWTPs) [3]. When antibiotics are consumed, they are often not completely metabolized in a human body; hence, they are excreted through urine and faeces, which are then transferred to WWTPs. Unfortunately, present-day wastewater treatment plants are not designed to fully remove these antibiotics. Thus, the effluent of WWTPs still contains antibiotic residues and their metabolites, which may also be pharmaceutically active, serving as a hotspot for facilitating the growth of antibiotic-resistant bacteria (ARB) via various mechanisms [4,5]. Other sources of antibiotic residues in the environment include untreated wastewater, run-off from agricultural activities, and discharge from aquaculture. The effluent from pharmaceutical industries that manufacture antibiotics and hospital waste can also contain a high load of these antibiotics, as illustrated in Figure 1. The occurrence of antibiotic residues in the environment has led to the spread of antimicrobial resistance, a global public health challenge and a leading cause of death [6,7].

**Figure 2:**
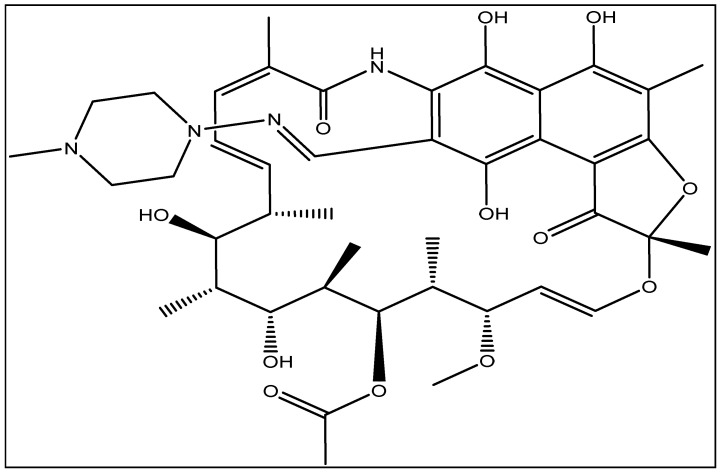
Chemical structure of rifampicin.

Rifampicin (RFA) (Figure 2) is a vitally important antibiotic used as the first line of treatment for tuberculosis caused by *mycobacterium tuberculosis*. Tuberculosis is the second deadliest infectious disease in the world, with the highest number of cases reported in Africa and Asia [8,9]. The continuous spread of this disease is worsened by the development of bacterial strains resistant to antibiotics commonly used for therapeutic treatment [10]. Recent studies have revealed the presence of RFA in the aquatic environment [11,12,13,14], and it has been associated with the spread of microbial resistance for the treatment of tuberculosis [15,16]. Furthermore, the presence of RFA in the aquatic environment increases the risk of its easy diffusion into the edible tissue of fishes due to its lipophilic nature, and thus poses various health challenges linked to human fish consumption [17]. It also increases the proliferation of antibiotic-resistant genes (ARGs) in pathogens affecting fish; alterations drive ecological change by affecting microbial assemblages [18]. Recently, the World Health Organization’s classification of antibiotic-resistant drugs highlighted RFA as an antibiotic of high priority [19]. Several methods have been employed for the removal of antibiotics from wastewater, such as advanced oxidation processes [20], membrane filtration [21,22], photocatalysis [23], the photo-Fenton process [11,24], and ion exchange [25]. Whilst these methods have proven to be effective at removing antibiotics, they have several limitations, such as technical complexity in operation, membrane fouling, the generation of toxic by-products, high costs of maintenance, and energy consumption [26,27].

Adsorption has proven to be one of the most effective methods for removing antibiotics from wastewater; it is eco-friendly and has a relatively simple mode of operation [28]. Several adsorbents have been investigated to remove RFA from wastewater. Barzegarzadeh et al. (2023) [29] examined the use of a copper-1,4-benzene dicarboxylic acid metal–organic framework attached to a wool bio-composite (Cu(BDC)@Wool) for the removal of RFA from wastewater through batch and fixed-column processes. This adsorbent can remove up to 99% of RFA under optimized conditions. Furthermore, ultrasound-assisted adsorption reduced the equilibrium time for RFA removal compared to conventional adsorption. Kais et al. (2020) [23] investigated the removal of RFA from wastewater using activated carbon made from cocoa shells, and the results showed that the adsorption capacity of RFA onto cocoa shells reached almost 80%. Shafaati et al. (2020) [30] developed a composite, GO/CS/Fe_3_O_4_, by synthesizing magnetized nanoparticles coated with chitosan (CS/Fe_3_O_4_) and grafted graphene oxide (GO) to remove RFA from aqueous solutions. This composite achieved more than 95% removal at pH 5 and 328 K. Lin et al. (2019) [31] investigated the simultaneous removal of both lead (Pb(II)) and RFA from wastewater using green-synthesized iron nanoparticles (Fe-NPs) with removal efficiencies of 97% and 68%, respectively. The cost-effectiveness of the adsorption process depends on the type of adsorbent, the preparation method, the amount of adsorbent, and the regeneration of the adsorbent [27]. Hence, there is still a need for a low-cost adsorbent that only requires a simple and cheap surface modification for effective removal [32].

Sawdust, a readily available lignocellulosic material obtained as a waste product from forestry and wood processing, has emerged as a promising low-cost material for wastewater treatment. Its composition, enriched with hydroxylic and carboxylic groups, makes it an effective adsorbent for removing organic pollutants [33]. Moreover, its adsorption capacity can be improved through simple and cost-effective acid or base treatments [34]. Alidadi et al. (2018) [35] used sawdust modified with different agents (CaCl_2_, NaHCO_3_, HCl, and FeCl_3_) for the removal of tetracycline from water. The results showed that sawdust modified with the FeCl_3_ solution was most effective in removing tetracycline from water, with a maximum removal rate of 98% at optimized conditions. To the best of our knowledge, this is the first study on using mahogany sawdust to remove RFA from water. This study aimed to design a low-cost material for removing RFA from water. The objectives of this study were as follows: (1) to investigate the effect of the chemical pre-treatment of sawdust on adsorption capacity; (2) to characterize the raw and treated sawdust using various techniques, including scanning electron microscopy, a CHNS analyser, a Mastersizer, Fourier Transform Infrared spectroscopy, and the pH point of zero charge (pH_pzc_); (3) to investigate the effect of various parameters on the efficiency of the sawdust materials, including contact time, adsorbent dosage, initial concentration, temperature, and pH; (4) to investigate the best solvent suitable for the regeneration of the sawdust materials.

## 2. Results and Discussion

### 2.1. Analytical Method

The UV spectrum scan of different concentrations of RFA showed that the wavelength of maximum absorbance was 333 nm. The calibration curve for RFA was constructed over a concentration ranging from 1.56 to 100 mg/L, and a strong linear correlation existed between absorbance and concentration, with a coefficient of determination (R^2^) of 0.9999. The precision of the method, expressed as percentage relative standard deviation (%RSD), was within the acceptable limit (<2.0%), indicating good reproducibility in determining RFA concentrations in water. The accuracy, expressed as percent recovery, is presented in Table 1, and reveals that the method is highly reliable and accurate for determining RFA concentration in water. The limit of detection (LOD) and limit of quantification (LOQ) for this method, which were found to be 1.31 mg/L and 3.97 mg/L, respectively, were obtained from Equations (3) and (4).

### 2.2. Characterization of Sawdust

**Figure 3:**
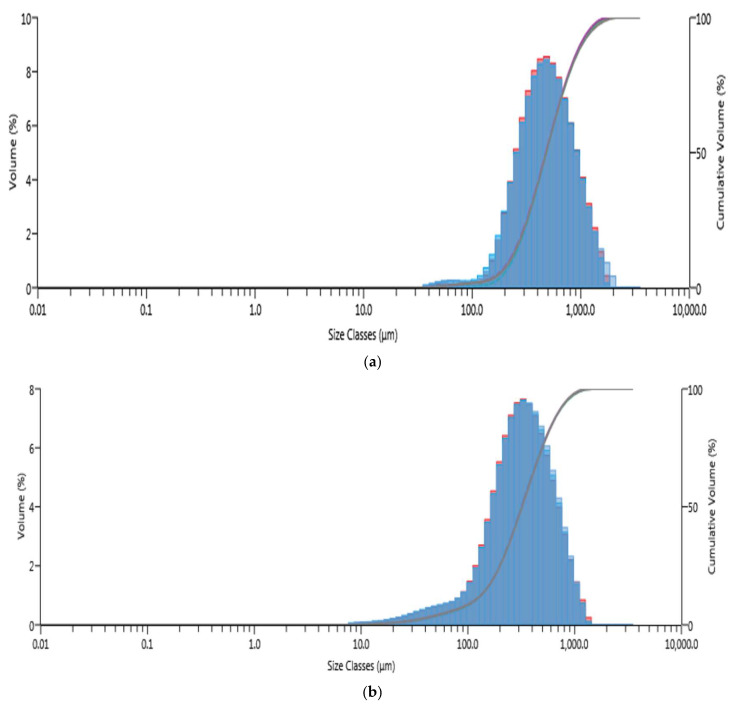
Particle size distribution of both sawdust types: (**a**) raw sawdust; (**b**) treated sawdust.

Particle size is a crucial characteristic of an adsorbent as it significantly influences the adsorption rate. The raw and treated sawdust samples exhibited a monomodal particle size distribution, indicating uniformity of the size distribution, as seen in Figure 3. This uniform distribution created consistent intraparticle pores that resulted in consistent diffusion paths for adsorbates, thereby enhancing the overall kinetics of the adsorption process. The volume-weighted mean and surface-weighted mean particle sizes of the raw sawdust were 560 µm and 403 µm, respectively, with a specific surface area of 0.015 m^2^/g. The area parameters (volume-weighted mean and surface-weighted mean) for treated sawdust were 360 µm and 190 µm, respectively, with a specific surface area of 0.031 m^2^/g, as shown in Table 2. The reduction in particle size following the acid treatment is attributed to the breaking down of inter- and intra-molecular bonds, which increased the surface area of the sawdust [36]. Table 2 also reveals the particle size distribution expressed as D values (percentiles). The D_50_ value represents the average particle size, while the D_10_ and the D_90_ values indicate the size below which 10% and 90% of the particles are found. The acid treatment resulted in a significant reduction in these D values, indicating a decrease in particle size. The efficiency of sawdust as an adsorbent is greatly impacted by particle size and surface area; as the particle size decreases, the specific surface area increases, enhancing the diffusion rate and increasing the adsorption capacity [37].

The CHNS analyses revealed the elemental composition of both the raw and treated sawdust, as presented in Table 3. The results elucidated that both types of sawdust have a high carbon content, as expected. There was no significant difference in elemental composition between the two types of sawdust, indicating that the acid treatment did not alter the composition. The high carbon content of both sawdust samples indicates their effectiveness in the adsorption of organic pollutants. Furthermore, the presence of hydrogen atoms, which are likely due to hydroxyl groups and other polar functional groups, enhances the polarity of the sawdust and influences its adsorption capacity. Interestingly, both types of sawdust possess low amounts of sulphur, suggesting that these materials have the potential to be sustainable energy sources [36,38].

**Figure 4:**
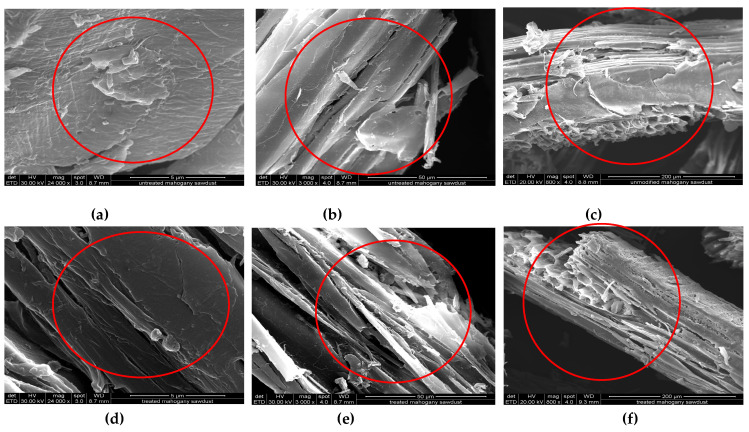
SEM images of both types of sawdust at different magnifications (mag): (**a**) raw sawdust at 24,000x; (**b**) raw sawdust at 3000×; (**c**) raw sawdust at 800×; (**d**) treated sawdust at 24,000×; (**e**) treated sawdust at 3000×; (**f**) treated sawdust at 800×. Circles highlight the areas of interest, illustrating the features described in the text.

Figure 4a–f shows the scanning electron microscopy (SEM) images of the raw and treated sawdust at different magnifications. The results showed that the surface morphology of the raw sawdust is rough, as illustrated in Figure 4a, while the surface of the treated sawdust is smooth, as seen in Figure 4d. This smoothness results from the acid treatment, which tends to break down the system of hydrogen bonding within the lignin structure. The smoothness of the surface of the treated sawdust helps the functional groups to become more easily accessible for adsorbates (i.e., RFA), making the treated sawdust a more efficient sorbent than the raw sawdust [39]. The fibres of the raw sawdust are still neatly organized and ordered, as revealed in Figure 4b, while for the treated sawdust, they are broken down into smaller fibrils, as seen in Figure 4e. Furthermore, Figure 4f shows that more surface pores were developed on the surface of the treated sawdust than the raw sawdust, as seen in Figure 4c. This is a result of the breakdown of the hydrogen bonding in the lignin surrounding the cellulosic fibres, and due to the dehydration of volatile compounds [40].

**Figure 5:**
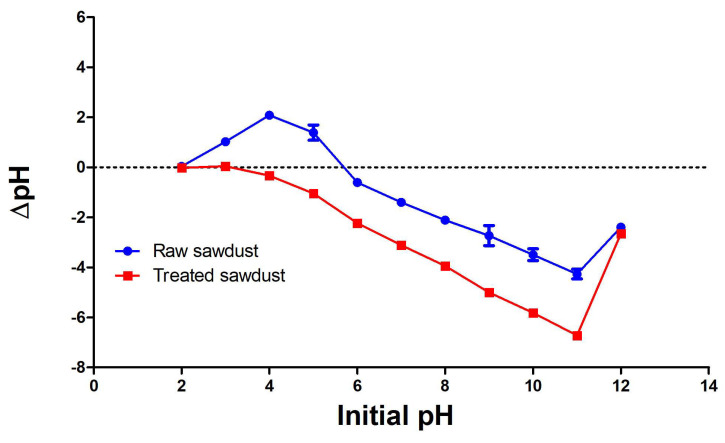
The values of pH_pzc_ of the raw and treated sawdust samples.

The pH point of zero charge (pH_pzc_) is the pH at which the net charge of the sawdust is zero. The pH_pzc_ values of the raw and treated sawdust adsorbents are 5.80 and 3.10, respectively, as presented in Figure 5. The results showed that the surface of both the raw and treated sawdust was acidic. However, the decrease in the value of the treated sawdust is due to the acid treatment, which further protonates the surface of the sawdust, thereby increasing its acidity. This is consistent with the results of Segovia-Sandoval et al. (2018) [41], who studied the removal of Zn(II) from an aqueous solution using treated walnut shells. The pHpzc for untreated walnut shells was 4.5, and 3.3 for walnut shells treated with citric acid. The acid treatment resulted in a 2.3 times increase in acidic sites on the treated walnut shells.

**Figure 6:**
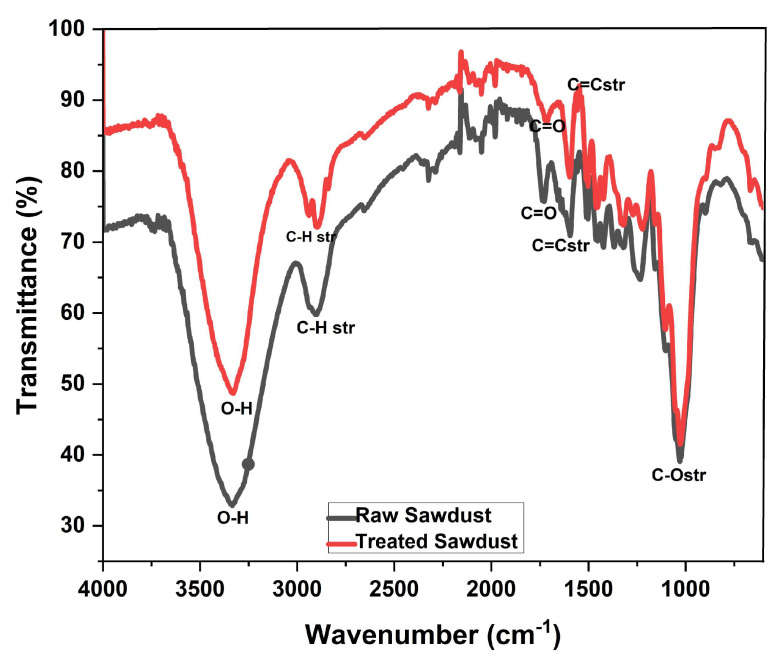
The FTIR-ATR spectra of the raw and treated sawdust materials.

Figure 6 shows the FT-IR spectra of both the raw and treated sawdust, indicating some functional groups responsible for the adsorption of antibiotics dissolved in water. Although there is great similarity between the raw sawdust and the treated sawdust, the strong and wide broad signal at the wavenumber of 3316 cm^−1^ (point A) indicates the presence of hydroxyl (-OH) groups. The signal around 2908 cm^−1^ (point B) aligns with the presence of C-H stretching; however, the increase in the intensity of this signal for the treated sawdust is due to the acid treatment [40]. The C band at 1708 cm^−1^ in the spectra of both the raw and treated sawdust corresponds to the carbonyl (-C=O) groups in the cellulose, hemicellulose, and lignin components of the sawdust, but the increase in the intensity observed for the treated sawdust can be explained by the breakdown of inter- and intra-molecular bonding during the acid-catalysed hydrolysis. The region on the FT-IR spectrum between the bands 1500 and 1000 cm^−1^ confirms the aromatic structures of the lignin component in the sawdust. The acid treatment disintegrates the intricate hydrogen bonding network within the cellulose, hemicellulose, and lignin constituents of the sawdust [36]. This disruption leads to the disappearance of peak D at 1342 cm^−1^ and an increase in intensity at other bands within this region, such as 1432 cm^−1^ and 1253 cm^−1^ in the treated sawdust.

### 2.3. Adsorption Study of RFA

#### 2.3.1. Effect of Contact Time

**Figure 7:**
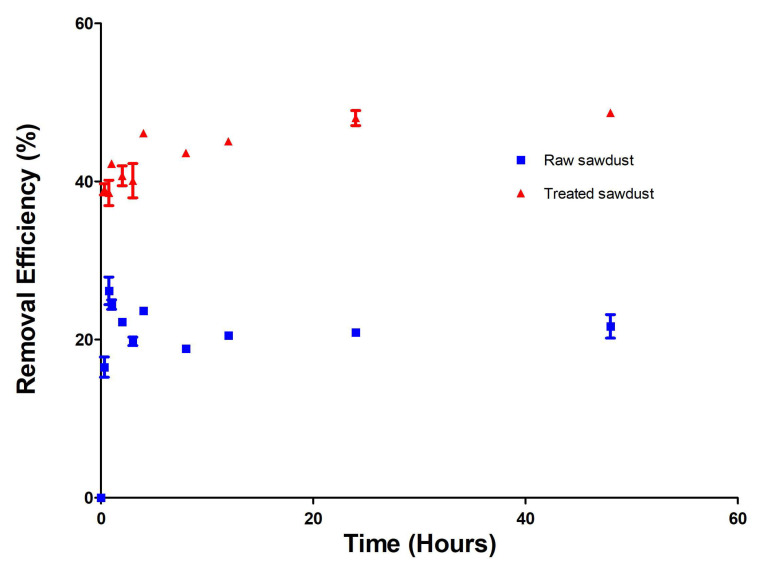
The effect of contact time on the removal efficiency of RFA from water by the raw and treated sawdust (mean ± standard error of the mean).

Figure 7 illustrates the removal efficiency of RFA by raw and treated sawdust over 48 h at room temperature. The effect of contact time on the removal efficiency was distinguished by three distinct phases. At the initial phase, between 0 and 45 min, the raw and treated sawdust had removed about 16% and 39% of RFA, respectively. The sudden increase in the removal efficiency within the first 45 min for both types of sawdust is attributed to the abundance of available adsorption sites. This is followed by a transitional phase where the adsorption rate slows down as the number of available sites decreases, with a slight decrease in RFA removal between 1 and 3 h for both the raw and treated sawdust. This is likely due to the desorption of weakly attached RFA molecules from the sawdust surface [42]. Finally, the adsorption system reaches equilibrium after 8 h, with a removal efficiency of about 20% and 45%, as seen by the plateau in Figure 7 due to the saturation of the surface groups of the raw and treated sawdust. These results agree with the findings of Kais et al. (2020) [23], who investigated the use of activated carbon derived from cocoa shells (ACCS) as an adsorbent for removing RFA from water. Their results showed an initial rapid uptake of RFA from water within the first few hours, gradually decreasing over time. The adsorption increased again until equilibrium was established following an extended contact period.

Moreover, the results also showed that the acid-treated sawdust had a higher removal efficiency compared to the raw sawdust because of its smaller particles sizes and increased surface area [35].

#### 2.3.2. Effect of Initial RFA Concentration

**Figure 8:**
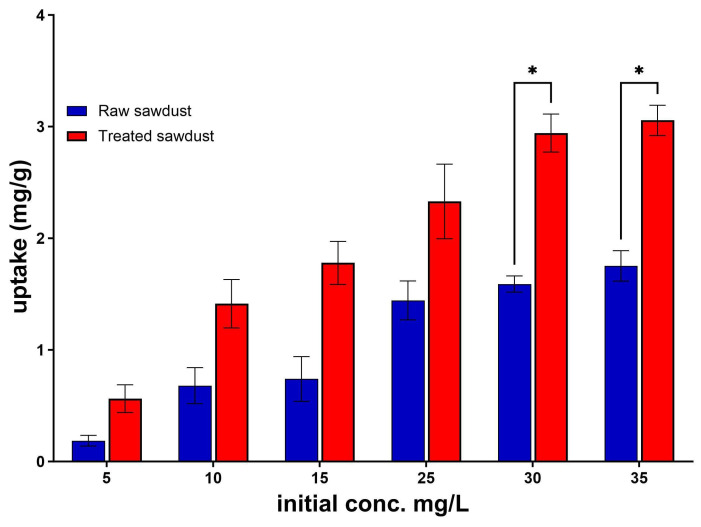
Effect of initial concentration on the uptake of raw and treated sawdust (mean ± standard error of the mean, *p* < 0.05 (*)).

The effect of the initial concentration of RFA on its uptake by both the raw and treated sawdust is presented in Figure 8. The results indicated that the uptake increased from 0.19 mg/g to 1.76 mg/g and from 0.56 mg/g to 3.05 mg/g as the initial concentration rose from 5 mg/L to 35 mg/L for raw sawdust and treated sawdust, respectively. This increase was a result of an increase in the driving force at a higher initial concentration, which moved the RFA molecules in the aqueous solution to the surface of the sawdust due to the higher concentration gradient, thus increasing the uptake as the initial concentration of RFA molecules per unit of volume increased [43]. These results align with the findings of Negarestani et al. (2023) [44], who studied the removal of RFA molecules from water using sisal–Layered Double Hydroxide (sisal-LDH), a bio-nanocomposite. Their results showed that the uptake of RFA increased from 11 mg/g to 38 mg/g as the concentration increased from 10 mg/L to 100 mg/L when the removal efficiency dropped from 99% to 10%.

#### 2.3.3. Effect of the Adsorbent Dosage

**Figure 9:**
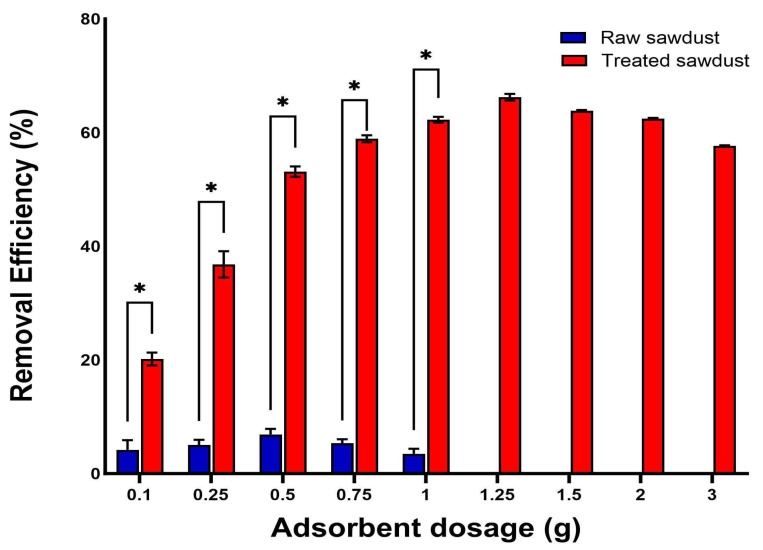
Effect of adsorbent dosage on the removal efficiency of RFA by raw and treated sawdust (mean ± standard error of the mean, *p* < 0.05 (*)).

The removal efficiency of RFA increased as the amount of raw sawdust and treated sawdust within the same volume increased until the optimal dose was reached, before declining, as shown in Figure 9. The optimal dose was where the maximum removal efficiency was achieved. This study’s optimal doses for the raw and treated sawdust were found to be 0.5 g and 1.25 g, respectively. The increase in the removal efficiency of RFA until the optimal dose was due to the increase in the number of active sites on the surface of the raw and treated sawdust; thus, the removal efficiency grew from 4 to 7% and from 20 to 65% for raw and treated sawdust, respectively. Further increases in the amount of sawdust beyond the optimal dose may lead to overcrowding of the active site and the unbinding of weakly attached molecules both on raw sawdust and treated sawdust, decreasing the removal efficiency [45]. The results are consistent with those published by Hamad et al. (2023) [46], who worked on the removal of levofloxacin from hospital wastewater using nano-zero-valent iron and nano-copper materials. Their results revealed that there was a continuous increase in removal efficiency until the optimal dosage, and beyond this point, there was a decrease in the percentage of levofloxacin removal. In another study, Movasaghi et al. (2019) [45] also reported similar trends for pretreated oat hulls used as an adsorbent to remove ciprofloxacin from water. Their findings showed that the maximum adsorption capacity was observed at a certain dosage and further increases in the dosage resulted in a decline in the adsorption capacity. Higher concentrations resulted in the overlapping of the adsorbent’s sites and the separation of weakly adsorbed molecules, which may have caused the decline in adsorption capacity.

#### 2.3.4. Effect of the pH of RFA Solution and Possible Mechanisms of Adsorption

**Figure 10:**
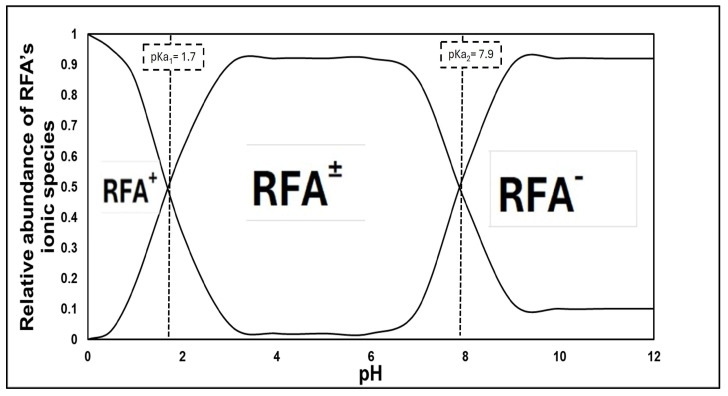
The ionic forms of RFA as a function of pH and pK_a_.

**Figure 11:**
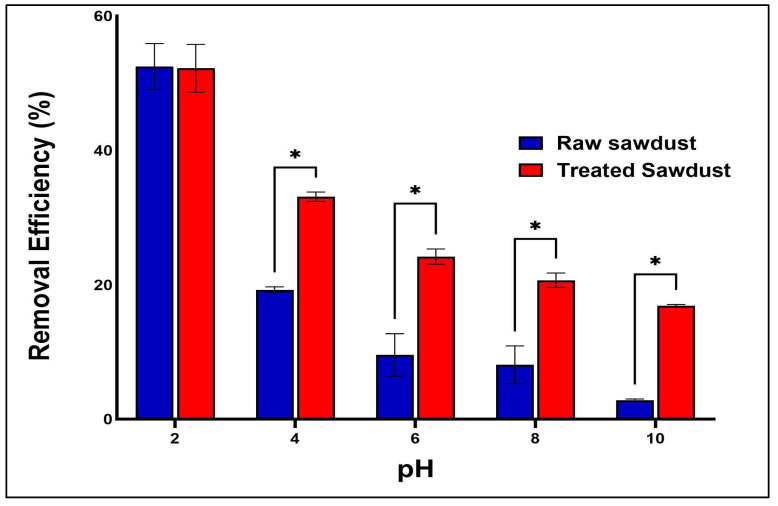
The effect of pH on the removal efficiency of RFA by raw and treated sawdust (mean ± standard error of the mean, *p* < 0.05 (*)).

**Figure 12:**
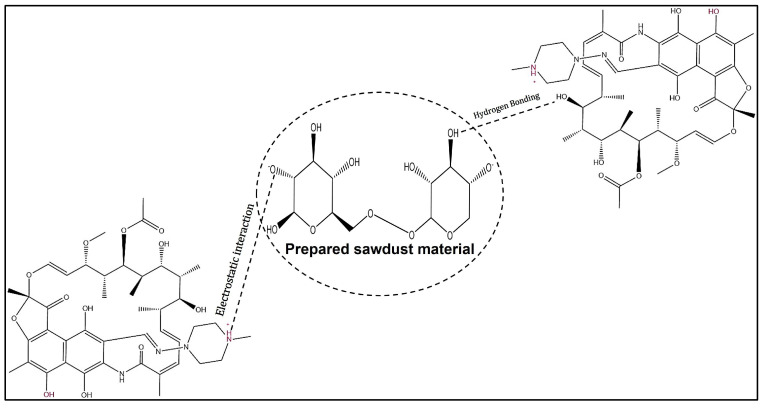
Graphical illustration of the adsorption mechanisms of RFA and both types of sawdust.

**Figure 13:**
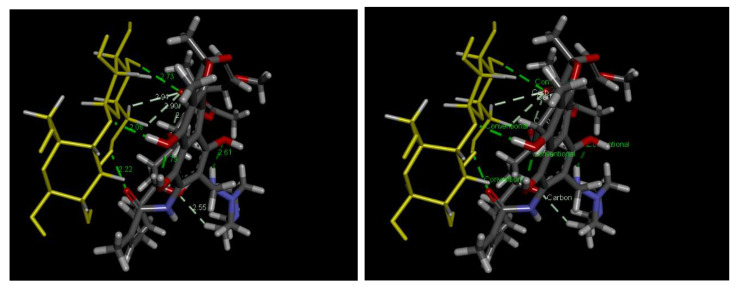
Three-dimensional structure of cellulose and rifampicin showing notable interactions at distances of 2.90 (carbon–hydrogen bond), 2.94 (carbon–hydrogen bond), 2.22 (conventional hydrogen bond), 2.06 (conventional hydrogen bond), and 2.73 (conventional hydrogen bond). (The overlapping point shows that there are various types of interaction occurring between the adsorbent (yellow molecule) and rifampicin molecule (Gray and black). For example, the green dotted line between the adsorbent molecule and the rifampicin represent formation of hydrogen bond (2.73) while white dotted line indicate the formation of carbon-hydrogen bond (2.94 and 2.90). The number indicate the bond length).

**Figure 14:**
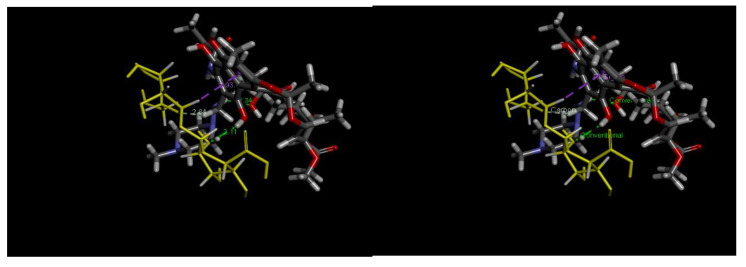
Three-dimensional structure of hemicellulose and rifampicin showing notable interactions at distances of 2.84 (carbon–hydrogen bond), 2.11 (conventional hydrogen bond), and 2.93 (Pi–sigma bond). (The overlapping point shows that there are various types of interaction occurring between the adsorbent (yellow molecule) and rifampicin molecule (Gray and black). For example, the green dotted line between the adsorbent molecule and the rifampicin represent formation of hydrogen bond (2.73) while white dotted line indicate the formation of carbon-hydrogen bond (2.94 and 2.90). The number indicate the bond length).

pH plays a critical role in the adsorption process, as it affects not only the surface charge on the sawdust, but also the ionized state of the antibiotics. Figure 10 shows the amphoteric nature of the RFA molecules at different pH ranges. As per the PubChem database (ID: 135398735), the pK_a_ values of RFA used were 1.7 (pK_a1_) and 7.9 (pK_a2_). Generally, RFA exists predominantly as a cation, where 3-piperazine nitrogen is protonated when the pH is below 1.7 (RFA^+^). A zwitterion is formed between the pH of 1.7 and 7.9 (RFA^±^), and an anion exists when pH > 8 due to deprotonation at the 4-hydroxyl group (RFA^−^) [18]. These transformations, presented in Figure 11, showed that the removal efficiency of RFA from water was maximal at acidic pH levels for both the raw and treated sawdust. However, the removal efficiency decreased as the pH increased from 2 to 10. The pH_pzc_ values of the raw sawdust and treated sawdust were 5.8 and 3.2, respectively. At pH below 2, both sawdust types were positively charged (pH < pH_pzc_), and RFA existed as a zwitterion; therefore, there was a high degree of electrostatic interactions between the sawdust particles and the RFA molecules. Furthermore, there was no significant difference in the removal efficiency of the raw and treated sawdust at a pH of 2. This is because, at pH 2, the raw sawdust surface may be altered by the highly acidic environment, thus making it act as a treated sawdust. As the pH increases, the surface charge on each sawdust type becomes negatively charged. When pH > pH_pzc_, the negative charge of the adsorbent became more pronounced, and also, at pH > pKa_2_, RFA exists as negatively charged ions, leading to higher electron repulsion forces between the antibiotic and the surface of the sawdust, resulting in a lower percent of its removal. These findings agree with the those obtained by Kais et al. (2020) [23], who reported that the removal efficiency of RFA from water was highest at a pH of 5–6. However, the removal efficiency decreased at pH levels above 6, with the adsorbent’s point of zero charge (pH_pzc_) being 6.8. This agrees with the work by Shafaati et al. (2020) [30], who reported that removal efficiency peaked between pH 3 and 5 and then declined as the pH increased from 6 to 9. The pH_pzc_ of the adsorbent used in their study was 5. Furthermore, the adsorption of RFA by both raw and treated sawdust may be explained by other mechanisms, such as π−π interactions and hydrogen bonding at higher pH levels where electron repulsion predominates (see Figure 12). This includes the π−π interactions between the RFA π bonds and the C=C bonds of the adsorbent. Furthermore, the deprotonated 4-hydroxyl groups can establish hydrogen bonds with the adsorbent’s −OH groups [43]. Furthermore, these interactions were further investigated using molecular docking simulations. The results revealed a strong hydrogen bonding interaction between rifampicin and the sawdust matrix (cellulose and hemicellulose) involving the hydroxyl and carbonyl groups, as shown in Figure 13 and Figure 14. The calculated binding energy of −2.7 kcal/mol for cellulose–rifampicin and −3.5 kcal/mol for hemicellulose interactions, respectively, indicates the formation of a strong interaction and a stable complex. This suggests that sawdust is a promising adsorbent for rifampicin removal from water.

#### 2.3.5. Effect of Temperature

**Figure 15:**
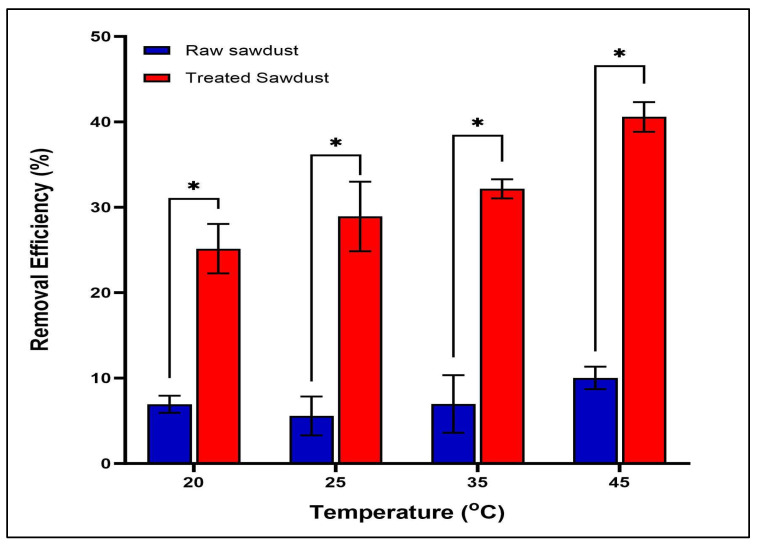
The effect of temperature on the adsorption of RFA by raw and treated sawdust (mean ± standard error of the mean, *p* < 0.05 (*)).

Temperature plays a critical role in the adsorption process of molecules onto the surface of the adsorbent. This is due to the diffusion rate of the adsorbate to the surface of the adsorbent. In this study, the effect of temperature was investigated at 20, 25, 35, and 45 °C, as shown in Figure 15. The removal efficiency by treated sawdust increased as the temperature increased, indicating that adsorption is an endothermic process. The raw sawdust did not show a significant difference in removal efficiency as temperature increased when the error bars were taken into consideration, although there was a slight increase at 45 °C compared to the levels measured at 20 °C. This indicates that adsorption processes by both types of sawdust were favourable at higher temperatures [44]. The increase in the removal efficiency at higher temperatures is due to the increase in the kinetic energy of the RFA molecules/ions, which accelerated from the solution volume faster to the available sites on the sawdust, thus increasing the removal efficiency. This result is in agreement with the one declared by Shafaati et al. (2020) [30], who studied the removal of RFA using a nanocomposite at a temperature range of 25 °C–55 °C. Their results showed that as temperature increased from 25 °C to 45 °C, the removal efficiency increased from 75 to 95%, indicating that the process is endothermic. The greater removal efficiency of the treated sawdust compared to the raw sawdust may be due to the acid treatment that altered the rough surface of the sawdust to a smoother surface. This makes the functional group more accessible, and hence as the temperature increases, the rifampicin molecules/ions move to the surface of the sawdust more quickly, facilitating an enhanced level of adsorption.

**Figure 16:**
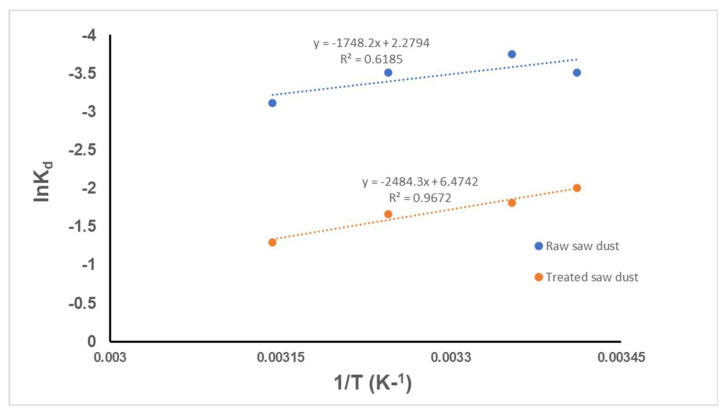
Dependence of InK_d_ on 1/T for RFA adsorption by the raw and treated sawdust.

Furthermore, the determination of thermodynamic properties helps us to understand the nature of the adsorption process and also the interaction of the adsorbate and the adsorbent [47]. In this study, free energy, enthalpy, and entropy were examined to explain the interaction between the RFA molecules and the sawdust. These were obtained from the experimental results using Von Hoff equations [29]:

(1) $$InK_{e}=\frac{-∆H}{R}\times \frac{1}{T}+\frac{∆S}{R\;}$$

(2) $$\mathrm{and}\;K_{e}=\frac{Q_{e}}{C_{e}}$$

where K_e_ is the thermodynamic equilibrium constant, T is the temperature of the solution in Kelvin (K), R (8.3144 J/molK) is the universal gas constant, and ΔH (KJ.${\mathrm{mol}}^{-1}$) and ΔS (J.${\mathrm{mol}}^{-1}.K^{-1}$) are changes in the enthalpy and entropy, respectively, and are obtained from the slope and intercept of the graph of lnK_d_ against 1/T, as shown in Figure 16 below.

The thermodynamic parameters for RFA adsorption by raw and treated sawdust are presented in Table 4 and Table 5, respectively. Both adsorption processes by the raw and treated sawdust have a positive value of change in enthalpy (ΔH), i.e., 14.35 KJmol^−1^ for raw (Table 4) and 20.65 KJmol^−1^ for treated (Table 5), indicating that both are endothermic processes. This agrees with the experimental results in Figure 13, which show that the removal efficiency of RFA increases as the temperature increases. A lower value ΔH, i.e., below 40 KJmol^−1^, indicates that the process is a physical adsorption process (physisorption) [29]. This suggests that the interaction between the raw and treated sawdust molecules is facilitated by electrostatic force, where the molecules or atoms adhere to the surface of the adsorbent through relatively weak intermolecular forces, such as van der Waals forces, dipole–dipole interactions, or hydrogen bonding (see Figure 12) [48]. These results are in agreement with Lin et al. (2019) [31], who studied the adsorption of RFA on iron nanoparticles and obtained a change in enthalpy of 23.08 KJmol^−1^, indicating that the adsorption process is endothermic and due to physical adsorption. The free Gibbs energies (ΔG) of the adsorption process for both raw sawdust and treated sawdust were negative, indicating that the adsorption process is spontaneous and favourable across all of the temperatures studied. The value of ΔG becomes more negative as the temperature increases for the raw and treated sawdust, agreeing with the observation that the adsorption process was enhanced at higher temperatures. This trend is in agreement with Ahsan et al., 2018 [43], who investigated the removal of tetracycline using treated sawdust. Furthermore, the Gibbs free energy change (ΔG) can act as a predictive indicator for the type of adsorption process, i.e., when ΔG falls within the 0–20 kJ/mol range, this signifies physical adsorption, and if ΔG lies between 80 and 400 kJ/mol, this indicates chemical adsorption [43]. Table 4 and Table 5 corroborate that both the raw and treated sawdust exhibit physical adsorption, which is consistent with the change in enthalpy (ΔH) results.

The entropy, S, of the adsorption process describes the state of disorderliness of the molecule from the aqueous solution to the surface of the adsorbent. The changes in entropy, ΔS, were 18.95 J mol^−1^ K^−1^ and 53.83 J mol^−1^ K^−1^ for the raw and treated sawdust, respectively. The positive value for both the raw and treated sawdust indicates that the degree of disorderliness of RFA molecules towards the surface of the adsorbent is smaller than the degree of randomness of RFA molecules in the surrounding aqueous solution [49,50]. Also, the higher value of ΔS for the treated sawdust system compared to the raw sawdust system indicates that more energy is required to detach the RFA molecule from the surface of the adsorbent [49]. This result also agrees with Shafaati et al.’s 2020 [30] study, where a positive value of entropy was obtained during the adsorption of RFA with a GO/CS/Fe_3_O_4_ composite [a magnetized nanoparticle coated with chitosan (CS/Fe_3_O_4_)-grafted graphene oxide (GO)].

### 2.4. Re-Usability of Sawdust

**Figure 17:**
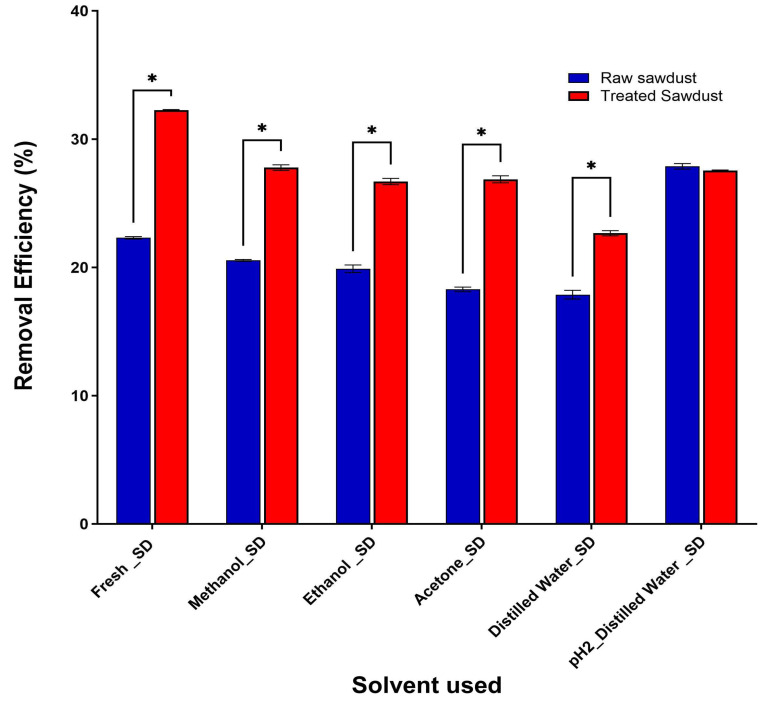
The effect of different solvents used for the regeneration of raw and treated sawdust. (mean ± standard error of the mean, *p* < 0.05 (*)).

The choice of solvent for regeneration is crucial as it can have significant environmental impacts and influence the overall cost. Figure 17 shows that regenerated raw sawdust with acidic water (pH 2) has the highest removal efficiency of 27%, even higher than the fresh sawdust. This is due to the pH of the acidified water affecting the surface of the raw sawdust, making more sites available for another adsorption process, while for the regenerated treated sawdust, both acidified water and other organic solvents have a similar removal efficiency. Nonetheless, using acidified water may be more favourable for the regeneration of both raw and treated sawdust because it is less toxic and cheaper than other organic solvents. This results aligns with the observation made by Alidadi et al., 2018 [35], that acidified water is a better eco-friendly and cheap regeneration solvent for sawdust. Although, their findings revealed that methanol-regenerated sawdust had a slightly higher removal rate than acidified water. Furthermore, Kwon et al., 2012 [51] regenerated phosphorylated sawdust to adsorb indium with 0.05 M HCl. The results revealed that the solvent had a minimal impact on the adsorption efficiency of the sawdust, with only a gradual decline of 2–4% per cycle after each regeneration. Although the solvent caused some reduction in efficiency, its effect was not severe enough to prevent the sawdust from being reused multiple times effectively.

## 3. Materials and Methods

### 3.1. Materials

The sawdust was sourced from a mahogany tree supplied by the Northern Ireland Environmental Agency. The antibiotic *RFA*$(C_{43}H_{58}N_{4}O_{12};molar\;mass\;833.5\frac{g}{mol}$; and purity of 97%) was purchased from Sigma Aldrich (London, UK) and characterized by limited solubility in water at 25 °C (2.5 mg/mL pH 7.3). Dimethyl sulfoxide (DMSO, purity of 99.9%, from Sigma Aldrich UK), sulfuric acid (H_2_SO_4_, 95 wt. % solution supplied by VWR Chemicals, Lutterworth, UK), hydrochloric acid (HCl, 37 wt. % solution supplied Scientific Laboratory Supplies, Nottingham, UK), and sodium hydroxide (NaOH) pellets from Scientific Laboratory Supplies UK, were used. Deionized water (DI) was used in the preparation of all solutions in this study, and all chemicals were used as received without any further purification.

### 3.2. Methods

#### 3.2.1. Method Development

The concentration of RFA in water was measured using a UV–visible spectrophotometer (Varian Cary 50 Bio model, Mason Technology, Dublin, Ireland). A stock solution of 100 mg/L of RFA was prepared and different working solutions with concentrations ranging from 0.39 to 100 mg/L were made from it. Each of the working solutions was scanned individually from 200 to 800 nm wavelength to determine the maximum absorption (λ_max_) for RFA. The λ_max_ obtained from these scans was used for all further UV-Vis measurements. A calibration curve was constructed by plotting the absorbance measured at (λ_max_) versus the concentrations of the calibrants. The method’s precision was estimated by calculating the relative standard deviation (% RSD) of repeated measurements at three different concentrations within the linearity range. The accuracy was determined by spiking RFA in blank water samples at three different concentration levels and calculating the recovery. The limits of detection (LOD) and quantification (LOQ) were determined based on the standard deviation of the response (σ) and the slope of the calibration curve (S), according to the following equations [52]:

LOD = 3.3(σ/S) (3)

LOQ = 10(σ/S)(4)

#### 3.2.2. Preparation of the Sawdust

The mahogany sawdust was washed thoroughly with warm distilled water to remove visible impurities and dried in an oven at 130 °C for 48 h. The dried sawdust was ground into fine particles and separated into different particle size fractions ranging from 38 to 850 μm by vibrating sieve shaking (Retsch AS200). The particle size in the range of 180–250 μm was selected for further processing because it has been shown to be most efficient for the adsorption process [4,36,53]. The sawdust fraction with the abovementioned particle size range was washed repeatedly with warm distilled water until the washing liquid ran clear. It was then vacuum-filtered and dried in an oven at 100 °C for 24 h to obtain the raw sawdust samples. Akinsanmi et al. (2018) [54] demonstrated that 2M sulphuric acid showed the highest adsorption potential and removal efficiency among the reagents used in their study. For chemical treatment, 50 g of the raw sawdust was immersed in 500 mL of 2 M sulphuric acid solution. This mixture was stirred continuously at 30 rpm, at room temperature, using a magnetic stirrer for 24 h. After 24 h of acid treatment, the slurry was transferred to an oven and heated at 60 °C for 2 h to facilitate further interaction. The acid-treated sawdust was vacuum-filtered and washed thoroughly with distilled water until a neutral pH was achieved in the washing filtrate. The washed sawdust was dried in an oven at 100 °C for 24 h to obtain the chemically treated sawdust. The samples were then used in the adsorption studies.

### 3.3. Characterization of Sawdust

The sawdust materials were characterized using various instrumentation techniques to determine the physiochemical and structural properties of the materials.

Scanning electron microscopy (SEM; FEI Quanta^TM^ 200 ESEM) is a technique that utilizes electron–material interactions to produce images of a sample’s surface. SEM was used to examine the surface morphology of both the raw and treated sawdust at different magnifications. A CHNS analyser was used to examine the constituents of raw and treated sawdust using a Perkin Elmer PE2400CHNS elemental analyser (Waltham, MA, USA). The particle size and particle size distribution of the selected fractions of sawdust were analysed using a Malvern Mastersizer 3000 (Malvern Panalytical, Worcestershire, UK). The surface functional groups present in the raw and treated sawdust samples were identified with the aid of Fourier Transform Infrared spectroscopy in Attenuated Total Reflectance (FTIR-ATR) mode using a Thermo Nicolet Nexus Spectrometer (Nicolet, Green Bay, WI, USA). Before the spectra collection, each sample was dried and ground using a pestle and mortar to ensure a good level of uniformity. Each sample of sawdust was placed on the diamond crystal, and the spectra were run (64 scans) over a wavenumber range of 4000–600 cm^−1^ with a resolution of 4 cm^−1^. The pH point of zero charge (pH_pzc_) was used to examine the surface chemistry of the sawdust. The pH_pzc_ is the pH at which the net charge of the sawdust is zero. This was determined by the immersion method, which involved adding 1 g of sawdust to 50 mL of 0.01 M solution of NaCl in different conical flasks. The pH of the NaCl solutions ranged from 2 to 12 (pH*_i_*), and the mixture was allowed to stand for 48 h. The mixture’s final pH (pH_f_) was then measured using the JENWAY pH meter. The pH_pzc_ was determined as the point of intersection of ΔpH (pH_f_-pH*_i_*) vs. the initial pH (pH*_i_*) graph with the *x*-axis [39].

### 3.4. Adsorption Experiments

The adsorption experiments were carried out in a batch process to investigate the effect of some operational parameters that affect the adsorption process. This study explored the impact of five operational parameters on the adsorption of rifampicin. These included the solution temperature (298–318 K), adsorbent dosage (0.1–3 g), contact time (0–48 h), initial concentration of RFA (5–35 mg/L), and pH (2–10). Throughout the adsorption experiment, the effect of a single parameter was investigated at a time, while maintaining all other parameters constantly. After each adsorption study, the adsorbent was filtered out using 0.45 µm filters, and the concentration of RFA was measured using a UV-Vis spectrophotometer. All experiments were carried out in triplicate and the removal efficiency and uptake were calculated using Equations (5) and (6), respectively:

(5) $$\mathrm{Removal}\;\mathrm{Efficiency}=(\frac{C_{O}-C_{t}}{C_{0}})\times 100$$

(6) $$\mathrm{Uptake}\;(\mathrm{mg}/g)\;q_{t}=\frac{\left(C_{0}-C_{t}\right)\;V}{m}$$

where C_o_ is the initial concentration of RFA(mg/L), C_t_ is the concentration of RFA at time t, m is the mass of adsorbent used (g), and V is the volume of RFA solution used (mL).

### 3.5. Re-Usability Study

The ability to reuse adsorbents is crucial in adsorption processes, as it helps to minimize operational costs. This study investigated using five different solvents to determine the most suitable one for regenerating the adsorbent. For this purpose, 0.2 g of RFA-loaded raw sawdust or treated sawdust was mixed with 50 mL of the one of the following solvents: methanol, ethanol, acetone, acidified water, deionized water, and alkaline water. The mixtures were shaken for 24 h at room temperature, filtered, washed to neutral pH values of the filtrate, and dried at 50 °C for 12 h. To assess the adsorption capability of the regenerated adsorbent, 0.1 g of raw sawdust or treated sawdust was reused in the adsorption of RFA from the solutions (initial concentration of 25 mg/L, pH 6.5, 20 °C). The duration of adsorption was 8 h; the removal levels were calculated and compared with the ones obtained with the fresh (unused) adsorbent.

### 3.6. Statistical Analysis

The statistical analysis was performed using GraphPad Prism version 10. The adsorption experimental results are expressed as mean ± standard error of the mean and presented in graphical form. The comparative analysis between the raw and treated sawdust adsorption results was undertaken using multiple independent t-tests followed by Holm–Sidak correction for multiple comparisons. Statistical significance was considered at *p* < 0.05 (denoted by *).

## 4. Conclusions

This study investigated the potential of a low-cost material (sawdust) as an adsorbent for the removal of residual rifampicin from water. Chemical pre-treatment of the sawdust with sulfuric acid resulted in a significant reduction in the median particle size, D_50_, from 490 µm to 360 µm, with a corresponding increase in the specific surface area from 0.0014 m^2^/g to 0.0031 m^2^/g. The SEM analysis also demonstrated that the surface morphology was substantially altered from a rough to a smoother surface, which enhanced the accessibility to the functional groups (e.g., -OH groups). The FTIR-ATR results showed similarity in the functional groups present in both the raw and treated sawdust, i.e., hydroxyl groups (-OH), carbonyl groups (-C=O), and -C=C- fragments. However, the treated sawdust was characterized by a higher intensity of the signals associated with these groups, thereby boosting the adsorption of RFA from water. Consequently, the removal efficiency of RFA significantly increased from 13% to 65% after the acid pre-treatment (at the adsorption dosage of 31.25 g/L and time of 8 h). The thermodynamic studies showed that the adsorption of rifampicin by raw and treated sawdust is endothermic and spontaneous across the studied temperature interval (293–318 K), but was more favoured at higher temperatures. The optimal pH for the adsorption process was found to be 2 for both the raw and treated sawdust and was attributed to the electrostatic interactions between the zwitterions of the rifampicin and the positively charged surfaces of both the raw and treated sawdust particles. The results also showed that, apart from electrostatic interactions, π–π interactions and hydrogen bonding are also involved in the adsorption process. Finally, the cost of various adsorbent materials varies significantly; according to Diagboya et al., 2020 [55], the cost of activated carbon manufactured commercially is USD 31.3 per Kg, while biochar production costs range from USD 0.24 to USD 20 per Kg, depending on the starting materials and production process [56,57]. Metal–organic frameworks (MOFs) are generally more expensive due to their complex synthesis process [58]. However, sawdust is an abundantly available waste material that can be easily modified with inexpensive and readily available chemical reagents. It was confirmed that sawdust can be effectively re-generated following treatment with acidified water. This research highlights the potential of using ubiquitous sawdust waste as a valuable adsorbent for the removal of residual antibiotics and other pollutants from water.

## Figures and Tables

**Table 1:** Results for accuracy (% recovery) and precision (%RSD) for the analysis of RFA in water.

Concentration, mg/L	Mean, mg/L	Standard Deviation	Recovery	% RSD
25.00	25.25	0.01	100.9–101.1 ± 0.05	0.05

**Table 2:** Particle size distribution of raw and treated sawdust.

	Volume-Weighted Mean D (4,3), µm	Surface-Weighted Mean D (3,2), µm	Specific Surface Area, m^2^/g	Dx10, µm	Dx50, µm	Dx90, µm
Raw Sawdust	560	403	0.0015	225	479	1010
Treated Sawdust	360	190	0.0031	114	318	704

**Table 3:** Elemental composition of raw sawdust and treated sawdust.

Sample	Carbon C %	Hydrogen H %	Nitrogen N %	Sulphur S %
Raw Sawdust	46.70	6.36	<0.3	<0.3
Treated Sawdust	47.87	6.77	<0.3	<0.3

**Table 4:** Thermodynamic parameters of RFA adsorption on raw sawdust.

T (K)	1/T (K^−1^)	K_d_ = Qe/Ce	InK_d_	ΔH (KJ mol^−1^)	ΔS (J mol^−1^ K^−1^)	ΔG^0^= ΔH − ΔS
293.15	3.41 × 10^−03^	2.98 × 10^−02^	−3.51	14.53	18.95	−5.54
298.15	3.35 × 10^−03^	2.36 × 10^−02^	−3.75			−5.64
308.15	3.25 × 10^−03^	3.00 × 10^−02^	−3.51			−5.83
318.15	3.14 × 10^−03^	4.46 × 10^−02^	−3.11			−6.01

**Table 5:** Thermodynamic parameters of RFA adsorption on treated sawdust.

T (K)	1/T (K^−1^)	K_d_ = Qe/Ce	InK_d_	ΔH (KJ mol^−1^)	ΔS (J mol^−1^ K^−1^)	ΔG^o^ = ΔH − TΔS
293.15	3.41 × 10^−03^	1.35 × 10^−01^	−2.01	20.65	53.83	−15.76
298.15	3.35 × 10^−03^	1.63 × 10^−02^	−1.82			−16.03
308.15	3.25 × 10^−03^	1.90 × 10^−01^	−1.66			−16.57
318.15	3.14 × 10^−03^	2.73 × 10^−01^	−1.30			−17.10

## Data Availability

Data can be made available upon request.
